# Unraveling the Janus-Faced Role of Autophagy in Hepatocellular Carcinoma: Implications for Therapeutic Interventions

**DOI:** 10.3390/ijms242216255

**Published:** 2023-11-13

**Authors:** Thi Ha Nguyen, Tuan Minh Nguyen, Dinh Thi Minh Ngoc, Taesik You, Mi Kyung Park, Chang Hoon Lee

**Affiliations:** 1 College of Pharmacy, Dongguk University, Seoul 04620, Republic of Korea; 2 Department of Cancer Biomedical Science, Graduate School of Cancer Science and Policy National Cance Center, Goyang 10408, Republic of Korea; 3 Department of Bio-Healthcare, Hwasung Medi-Science University, Hwaseong-si 18274, Republic of Korea

**Keywords:** hepatocellular carcinoma, autophagy, mitophagy, autophagy inhibitors, autophagy inducer

## Abstract

This review aims to provide a comprehensive understanding of the molecular mechanisms underlying autophagy and mitophagy in hepatocellular carcinoma (HCC). Autophagy is an essential cellular process in maintaining cell homeostasis. Still, its dysregulation is associated with the development of liver diseases, including HCC, which is one of leading causes of cancer-related death worldwide. We focus on elucidating the dual role of autophagy in HCC, both in tumor initiation and progression, and highlighting the complex nature involved in the disease. In addition, we present a detailed analysis of a small subset of autophagy- and mitophagy-related molecules, revealing their specific functions during tumorigenesis and the progression of HCC cells. By understanding these mechanisms, we aim to provide valuable insights into potential therapeutic strategies to manipulate autophagy effectively. The goal is to improve the therapeutic response of liver cancer cells and overcome drug resistance, providing new avenues for improved treatment options for HCC patients. Overall, this review serves as a valuable resource for researchers and clinicians interested in the complex role of autophagy in HCC and its potential as a target for innovative therapies aimed to combat this devastating disease.

## 1. Introduction

Liver cancer stood as one of the leading causes of cancer-related fatalities in 46 coun-tries [1], resulting in over 700,000 annual deaths [2]. In the year 2020 alone, there were 905,700 documented cases of liver cancer worldwide, with 830,200 individuals succumbing to the disease [1]. It is anticipated that there could be an increase of over 55% in both new cases and deaths from liver cancer by the year 2040 [1]. Among primary liver cancers, hepatocellular carcinoma (HCC) dominates, accounting for roughly 90%, followed by in-trahepatic cholangiocarcinoma and other primary liver malignancies [3]. Notably, nearly 90% of HCC can be traced back to known underlying causes, with chronic viral hepatitis, heavy alcohol consumption, and non-alcoholic fatty liver disease being the most prevalent culprits [4].

Autophagy is a multi-step catabolic process that occurs in cells at a basal level and is triggered by various stressors that disrupt cellular homeostasis. These stressors can include low levels of cellular ATP, nutrient and growth factor deficiencies, hypoxia, endoplasmic reticulum (ER) stress, pathogen entry, or exposure to anticancer drugs [5,6]. There are three major forms of autophagy: macroautophagy, microautophagy, and chaperone-mediated autophagy. Among these, macroautophagy is commonly referred to simply as autophagy due to its better-known nature, while the other forms are still being explored [7]. In addition to the three primary forms of autophagy, there are various selective autophagy processes. For instance, mitophagy is a selective form of autophagy that specifically targets and isolates damaged or dysfunctional mitochondria for degradation. Another example is ER-phagy, which targets specific regions of the ER for degradation [8].

During this process, double membrane autophagosomes envelop misfolded proteins and damaged organelles. They subsequently merge with lysosomes, leading to the breakdown or recycling of their contents, including vital cellular components. This recycling sustains the creation of new materials and aids in maintaining cellular balance [9]. As a result, the primary role of autophagy is to promote cell survival during stressful situations.

However, it is essential to note that autophagy can also have a dual nature in cancer [6]. While it predominantly facilitates cell survival, overactivation of autophagy can lead to a form of cell death known as autophagy-dependent cell death. This phenomenon has been observed in contexts where excessive autophagy contributes to cell death [6]. Consequently, understanding the molecular mechanisms and physiological roles of this alternative non-apoptotic programmed cell death is crucial.

Given its intricate role in cellular processes, autophagy has emerged as a potential therapeutic target for various diseases, including neurodegenerative disorders and cancers like HCC [7,8,10]. Nevertheless, further research is needed to fully comprehend the mechanisms underlying non-apoptotic programmed cell death and its implications for potential therapeutic interventions.

Defective autophagy in cancer cells is increasingly recognized for its role in genomic damage, metabolic stress, and tumor development. Many studies have linked autophagy to cancer initiation, progression, and potential therapies. Intriguingly, some research indicates that autophagy can influence pro-oncogenes, promoting tumorigenesis and cancer progression [11,12]. Some studies indicate autophagy can suppress cancer [13,14]. Autophagy’s role in HCC varies depending on factors like cancer stage and pathology. Caution is needed in targeting autophagy for HCC treatment, and further research is required to understand its exact mechanisms.

Previous reviews have highlighted the roles of autophagy in HCC progression and therapeutic approaches [13,15,16,17]. In contrast, our review aims to incorporate the most recent discoveries in autophagy regulation, particularly focusing on selective autophagy like mitophagy, and their impact on the development and progression of HCC. Additionally, we explore the potential of modulating autophagy (either inhibiting or activating it) to enhance the effectiveness of cancer treatment, whether through traditional therapies or immunotherapy combinations.

Our review also underscores the significance of autophagy-related genes as potential biomarkers for HCC prognosis. Furthermore, as the importance of neuroscience in cancer research is increasingly recognized, we introduce the role of autophagy in the neural input aspects of HCC and discuss its potential implications.

## 2. Mechanism of Autophagy

The autophagy process is tightly regulated by a complex molecular machinery encoded by approximately 40 different autophagy-related genes (*Atg*) [18,19]. These genes were initially identified through genetic screens in yeast and have been extensively studied under various physiological and pathological conditions. The core autophagy pathway is highly conserved from yeast to humans. Numerous previous reviews have described the molecular machinery involved in mediating autophagy.

Autophagy is a well-studied multi-stage and dynamic process that involves the formation of autophagosomes and autolysosomes. It includes several distinct steps: induction, vesicle nucleation, lysosome fusion, and degradation. To initiate autophagy, the ATG1/Unc-51-like autophagy activating kinase 1 (ULK1) autophagy-related kinase complex is required [20]. This complex transduces signals to regulate the activity of the class III phosphatidylinositol 3-kinase PI3K/Ptdlns(3)K complex [21]. This complex is composed of several proteins, including Vacuolar Protein Sorting 34 Homolog (VPS34), VPS15, ATG6 (Beclin1), and ATG14. Together, they are required to form phosphatidylinositol 3-phosphate (PI3P) molecules on cellular membranes, which facilitates the recruitment of other proteins to the autophagosomal membrane [5]. 

During the elongation and expansion of the phagophore membrane (step 2), ER resident transmembrane protein 41B mobilizes lipids to the emerging autophagosome membrane [22,23]. Here, ATG2A delivers lipids to the PI3P-WD repeat domain phosphoinositide interaction (WIPI2) complex, and ATG9 rearranges lipids into a symmetrical bilayer that feeds the autophagosome [24]. ATG7 and ATG3 act as E1- and E2-like enzymes, respectively, to facilitate the conjugation of ATG8 (LC3) proteins to phosphatidylethanolamine. Then, the ATG12-ATG5-ATG16L complex functions as an E3-like enzyme, ultimately catalyzing the binding of LC3-I to PE leading to forming LC3-II [25]. LC3-II is associated with mature autophagosomes and is commonly used as a marker to analyze autophagic activity by assessing the number and distribution of autophagosomes.

**Figure 1:**
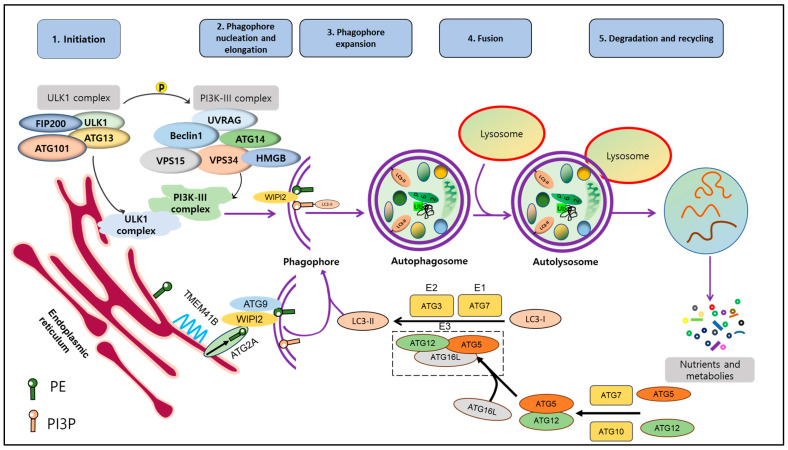
Molecular mechanism of autophagy. Autophagy process includes 5 stages: induction, nucleation, expansion, fusion, degradation and recycling. Upon the activation of autophagy, ULK1 complex (FIP200, ULK1, ATG101, ATG13) is activated and phosphorylated to facilitate formation of PI3K-III complex. After that, interaction between these complexes initiates phagophore formation. Besides that, free LC3 is cleaved by ATG4 into LC3-I, which is conjugated to PE by the assistance of ATG7 and ATG3 as well as ATG12-5-16L1 complex to ultimately form LC3-II. LC3-II and complex ATG12-5-16L1 facilitate induction and nucleation of phagophore. LC3-II binds to membrane of phagophore and exists during autophagy cascade until the final step. Damaged proteins and organelles, ubiquinated-p62 are enclosed in phagophore and further autophagosome. Autophagosome then is fused with lysosome to become autolysosome. Lysosomal hydrolases support degradation to create nutrients and materials, which are essential for maintaining cell survival. This figure is modified from Hashemi et al. [13]. Abbreviations: autophagy-related protein (ATG101, ATG12, ATG13, ATG16L, ATG3, ATG7), 200 kDa FAK Family Kinase-Interacting Protein (FIP200), High-mobility group box (HMGB), Microtubule-associated proteins 1A/1B light chain 3 (LC3), Sequestosome 1 (p62), Phosphatidylethanolamine (PE), Phosphatidylinositol 3-phosphate (PI3P), Transmembrane protein 41B (TMEM41B), Unc-51-like kinase 1 (ULK1), UV radiation resistance-associated gene (UVRAG), Vacuolar protein sorting (VPS15, VPS34), WD repeat domain phosphoinositide-interacting protein 2 (WIPI2).

Finally, autophagosomes fuse with lysosomes with the help of Rab proteins and the SNAP receptor machinery, forming autolysosomes. In autolysosomes, cellular components are degraded or recycled by the action of hydrolytic enzymes present in this compartment [26] (Figure 1).

## 3. Autophagy-Regulating Signaling Pathways in HCC

Regarding cellular processes, autophagy holds a central role, governing the self-cleansing and recycling mechanisms vital for cellular health. At the core of this autophagic regulation are several key signaling pathways, including phosphoinositide 3 kinase (PI3K)/AKT/mammalian (or mechanistic) target of rapamycin (mTOR), Adenosine monophosphate-activated protein kinase (AMPK)/mTOR, the mitogen-activated protein kinases (MAPKs), and Nuclear factor erythroid 2-related factor 2 (Nrf2)-p62. These pathways play a critical role in regulating autophagy, influencing whether cells undergo self-preservation or self-destruction. We give an insight into intricate workings of these signaling pathways, exploring their profound impact on autophagy and their essential roles in HCC development.

### 3.1. PI3K/AKT/mTOR

The PI3K/AKT/mTOR pathway plays a crucial role in HCC cell survival, as it is essential for maintaining high metabolism of glucose, lipids, and proteins in malignant liver cells [27]. Genomic studies in HCC patients have indicated that the mTOR pathway and its upstream signals, PI3K and AKT, are major players in the deregulated pathways in HCC. Global transcription analyses of 57 HCC tumors revealed that approximately 50% were associated with activating the Wnt or AKT pathways [28,29]. Furthermore, a meta-analysis of eight datasets, with different subgroups based on clinical parameters such as tumor size and Alpha-fetoprotein (AFP) levels, showed that subclass S2 was characterized by AKT activation in combination with MYC [30,31]. 

The PI3K/AKT/mTOR signaling pathway has garnered extensive attention as a critical modulator of autophagy [32]. When mTORC1, the central checkpoint of this pathway, is active, either through its upstream activation by PI3K/AKT [33,34] or through AMPK inhibition, the autophagic machinery is shut down [35,36,37].

Comparative analyses of gene and protein expressions of mTOR and LC3 in HCC and colorectal liver metastasis tissues have been conducted. The results indicated that mRNA expression of mTOR was significantly lower in metastatic tissues compared to HCC. However, protein levels of phosphorylated mTOR (p-mTOR) and LC3 were significantly higher in colorectal metastases compared to HCC [38]. 

The modulation of autophagy is influenced by growth factors or proliferation cytokines, such as insulin-like growth factor-1 and interleukin 3, which activate the PI3K pathway and phosphorylate AKT at serine residue 473, leading to the inhibition of autophagy [39,40,41]. Conversely, under stress conditions like insufficient nutrition and a hypoxic microenvironment, this pathway is inactivated, resulting in the induction of autophagy and suppression of cell growth and proliferation. Inactivation of mTORC1 leads to the dephosphorylation of autophagy/Beclin1 regulator 1 [42], activation of ULK1 complex, and initiation of autophagosome formation [43,44]. Polo-like kinase 1 negatively regulates MTORC1, activating autophagy [45,46]. 

**Figure 2:**
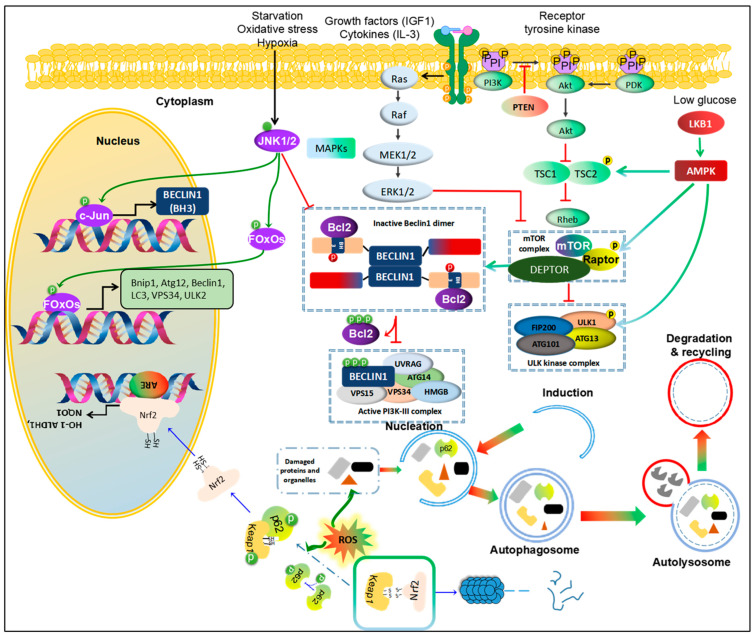
Pathways regulating autophagy in HCC. 1. PI3K/Akt/mTOR and MAPK/mTOR. Growth factors and cytokines activate receptor tyrosine kinase, followed by activating PI3K/Akt and MAPK pathways. PTEN inhibits phosphorylation of PI3K to block PI3K/Akt signal. With these agents, PDK also is promoted to recruit to activate Akt. Activated Akt inhibits the complex TCS1/TCS2, leading to enhancing Rheb activity. Further, Rheb directly binds to mTOR to activate mTOR complex, resulting in inhibition of ULK1 complex or enforcing the binding between Beclin1 and Bcl-2, and consequently blockage initiation of autophagy. By contrast, Ras/Raf/MEK/ERK pathway inhibits mTOR activity to enhance autophagy. 2. MAPK/JNK. Activation of JNK1/2 promotes autophagy in starvation, oxidative stress and hypoxic conditions. JNK1/2 directly enhances transcription of c-Jun via phosphorylation of c-Jun to increase Beclin1 expression. Moreover, JNK phosphorylate FOxOs, a transcription factor, to increase expression of autophagic proteins, like ATG12, Beclin1, LC3, Vsp34, ULK2 and Bnip1. Phosphorylated JNK also facilitates release Bcl-2 from Beclin1 to form active PI3K-II complex. 3. AMPK/mTOR. A low-glucose condition activates LBK1/AMPK to promote autophagy via phosphorylation of TCS1/2, RAPTOR or ULK1. 4. Nrf2-Keap1-p62. Normally, Keap1 binds to Nrf2 to drive into degradation of Nrf2 by proteasome. However, in response to ROS accumulation, autophagy could be promoted to clear the damaged proteins and organelles as well as decrease p62 levels. If this pathway is blocked, accumulated p62 would be phosphorylated to bind to Keap-1 and release free Nrf2. Then, Nrf2 is translocated into nucleus and binds to ARE promoter to enhance transcription genes against an oxidative condition. This figure is modified from Sun et al. [52]. Abbreviations: Protein kinase B (Akt), AMP-activated protein kinase (AMPK), B-cell lymphoma 2 (Bcl-2), Bcl-2/adenovirus E1B 19 kDa protein-interacting protein 1 (Bnip1), Transcription factor AP-1 (c-Jun), DEP Domain Containing MTOR Interacting Protein (DEPTOR), Forkhead box O (FOxOs), c-Jun N-terminal kinases 1 and 2 (JNK1/2), Kelch-like ECH-associated protein 1 (Keap1), Liver kinase B1 (LKB1), Mechanistic target of rapamycin (mTOR), Phosphatase and tensin homolog (PTEN), Regulatory associated protein of mTOR (RAPTOR), Tuberous sclerosis (TSC1 and TSC2).

Suppressor of cytokine signaling 5 overexpression can promote cell invasion and migration by inhibiting the PI3K-Akt-mTOR-regulated autophagy in HCC in vitro [47]. Another study supports the role of autophagy in HCC, where lncRNA DCST1-AS1 acts as a carcinogen and AFP as a key regulator of cell viability in HCC, promoting cell viability and invasion while inhibiting autophagy by mediating the AKT/mTOR signal transduction pathway [48,49]. The inhibition or mutation of Phosphatase and tensin homolog (PTEN), a tumor suppressor, is associated with the activation of the AKT-PI3K-mTOR pathway, promoting the initiation and progression of HCC [50]. Treatment of HCC with natural compounds, such as Cucurbitacin B, activates the phosphorylation of PTEN and further inhibits the expression of the AKT/mTOR oncogenic pathway while inducing a protective form of autophagy [51]. This observation suggests that autophagy could play a pro-oncogenic role through the PTEN-mTOR pathway (Figure 2).

### 3.2. AMPK/mTOR

AMPK plays a crucial role in maintaining energy balance within the cell. It exerts two major actions: inhibiting synthetic pathways while activating various catabolic processes, including glucose metabolism and autophagy [53]. During stress conditions that lead to ATP reduction, the energy-sensing kinase liver kinase B1 activates AMPK [54]. Once activated, AMPK can phosphorylate TSC2 and Raptor (the regulatory-associated protein of mTOR), resulting in the inactivation of mTORC1 and the induction of autophagy [55]. Moreover, AMPK can be activated by glucose starvation, leading to promoting autophagy through direct phosphorylation of ULK1 or other autophagy core components such as ATG9, VPS34, and Beclin1 [56,57,58,59,60]. This activation of autophagy is one of the mechanisms by which AMPK helps cells adapt to energy stress and maintain cellular homeostasis.

Recent studies have highlighted the role of AMPK-dependent autophagy in HCC. The activation of AMPK has been shown to inhibit migration and invasion and promote apoptotic cell death in HCC cells [61,62]. Additionally, AMPK activation has been identified as a critical predictive factor in tumorigenesis for patients with liver cirrhosis, as those with lower levels of phosphorylated AMPK (Thr172) had a significantly higher occurrence rate of HCC. Furthermore, blocking autophagy using chloroquine (CQ) eliminated the protective role of phosphorylated AMPK [63].

In HCC, the overexpression of genes such as *Sox18* or *SCD1* promoted cell proliferation, migration, invasion, and epithelial-to-mesenchymal (EMT). Conversely, the downregulation of these genes suppressed cell survival and metastasis, leading to activation of autophagy through the AMPK/mTOR signaling pathway [64]. Moreover, glycochenodeoxy cholate, a significant component of bile acid, was found to promote the invasion and migration of HCC by inducing autophagy through the activated AMPK/mTOR pathway [65]. HMGB1 also promotes doxorubicin resistance in HCC by inducing autophagy through the AMPK/mTOR signaling pathway [66].

The AMPK/cAMP- cAMP response element-binding protein (CREB) signal activated by metformin enhanced the expression of CCAAT/enhancer binding protein delta, which facilitated autophagic and apoptotic cell death while also resensitizing HCC cells to sorafenib [67]. Nucleotide-binding oligomerization domain 2 (NOD2) overexpression or CXCL17 knockdown NOD2 exhibited anti-tumor characteristics by activating the AMPK pathway through direct binding with the AMPKα-LKB1 complex, resulting in the increased autophagy-modulated apoptosis of HCC cells [68,69]. 

A loss of AMPK function may enhance cell progression, proliferation, migration, and invasion through various oncogenic molecules and pathways downstream of AMPK, indicating its potential anti-HCC function [70]. Conversely, some activators, like S-Phase kinase-associated protein 2, can upregulate the AMPK-ERK/CARM1 transduction pathway during the formation of liver cancer, suggesting that an over-activated autophagy in tumor tissues could contribute to HCC development [71,72] (Figure 2).

### 3.3. The MAPK (JNK, p38 and ERK)

Based on their structure and function, MAPK can be mainly divided into three groups: (1) c-Jun N-terminal kinase or stress-activated protein kinase (JNKs), (2) extracellular signal-regulated kinases (ERKs), and (3) the p38 MAPK [73]. The MAPK/ERK signaling transduction pathway was activated in 58% of the cases of HCC. It showed a positive correlation with the tumor size of HCC. Additionally, MAPK/ERK signaling may play an essential role in multi-stage hepatocarcinogenesis, at least partly through the upregulation of cyclin D1 [74].

The RAF-MEK-ERK axis plays a critical role in regulating various cellular processes, including cell proliferation, differentiation, angiogenesis, and survival of HCC cells [75]. Hepatitis B virus (HBV) and hepatitis C virus (HCV) can exploit this cascade to promote hepatocyte survival and viral replication by inducing autophagy [76]. The RNA-binding protein PNO1 regulates autophagy and apoptosis in HCC through the MAPK signaling pathway [77]. Moreover, there is a crosstalk between the p38MAPK pathway and autophagy-related genes, which may modulate mitochondria-mediated apoptosis in response to anti-Fas antibody/actinomycin in HCC cells [78]. As a result, MAPK has emerged as a potential target for HCC treatment.

In recent studies, a novel platelet-derived growth factor receptor α inhibitor was found to induce cell death through autophagy activation via the MAPK pathway in HCC cells [79]. Additionally, JNK activation has been shown to induce cell death of HT-29 and Caco-2 human colon cancer cells through autophagy, involving upregulation of mRNA levels of genes related to autophagy (*Bnip1*, *Atg12l*, *Beclin1*, *Lc3*, *Vsp34*, *Ulk2*) via the interaction between JNK and FOXO [80,81].

Myricetin induces autophagy and cell cycle arrest in HCC by inhibiting the membrane-associated RING-CH finger protein 1 (MARCH1)-regulated Stat3 and p38 MAPK signaling pathways [82]. β-Thujaplicin, a tropolone derivative, induces autophagic cell death, apoptosis, and cell cycle arrest in human HCC through ROS-mediated Akt and p38/ERK MAPK signaling pathways [83].

### 3.4. Nrf2-p62 Pathway

Recently, the Nrf2-p62 mechanism has emerged as an important regulator of autophagy activation in response to oxidants and redox stressors [84]. This mechanism involves the phosphorylation of p62, which increases its affinity for the Nrf2 binding site on Kelch-like ECH-associated protein 1 (Keap1), which is an adaptor for E3 ubiquitin ligase; through this Nrf2 is constantly degraded by proteasome. Additionally, when Keap1 binds to p62 and sequesters it within p62-bodies, Nrf2 avoids degradation and becomes activated [85]. As a result, p62 binding to Keap1 weakens the Keap1-Nrf2 interaction, releasing Nrf2 into the cytoplasm. Especially, p62 can sequester ULK1-mediated serine 349 phosphorylation of Keap1, leading to the redox-independent activation of Nrf2 [86]. Accumulated cytosolic Nrf2 then migrates to the nucleus. It attaches to specific sequences called antioxidant/electrophile responsive elements, activating the transcription of genes involved in the cellular response to oxidative stress [84].

Additionally, p62 is degraded by autophagy, demonstrating the correlation between the regulation of autophagy and the Nrf2 pathway [87]. Defective autophagy, leading to the accumulation of p62, can activate the Nrf2 pathway and promote the initiation of liver tumors in mice [88]. Conversely, inactivation of the Nrf2 cascade in the early stages of tumorigenesis may contribute to the enhanced tumorigenic potential of early hepatic lesions due to a reduced capacity for detoxification and cytoprotective processes. Thus, defective autophagy in liver cells may play a crucial role in tumor formation.

In HCC progression, activated Nrf2 appears to persist due to ROS sustained metabolism and flux. This activation of Nrf2 serves as a key stimulus for promoting cancer-associated autophagy, providing substrates for the energy and intermediary metabolism of cancer cells [89].

Therefore, the p62-Keap1-Nrf2 axis appears to play a crucial role in regulating various homeostatic mechanisms in the liver. Recent evidence has shown that in autophagy-deficient livers, the activation of this axis can reprogram HCC cell metabolism, leading to tumor growth, evasion of apoptosis, and drug resistance [90]. Moreover, Nrf2 protects cancer stem cells from damage caused by ROS, supporting tumor growth and providing resistance against chemotherapy and radiotherapy. Additionally, Nrf2 participates in the differentiation of cancer stem cells by regulating the autophagy process [91,92]. 

Currently, targeting p62 or the Keap1/Nrf2 system and related pathways, such as autophagy, holds promise as a potential therapeutic strategy in HCC therapy [84]. More detailed molecular mechanisms underlying the therapeutic perspective of targeting Nrf2-p62 via autophagy in HCC have been discussed and reviewed [84].

### 3.5. Crosstalks between These Pathways

The PI3K/AKT/mTOR pathway crosstalks with the AMPK/mTOR pathway through the TSC1/TSC2 molecules, regulating mTOR activity [55]. Specifically, AKT inhibits the activity of TSC1/TSC2, while AMPK promotes their activity through phosphorylation. Additionally, the MAPK pathway, including JNK, p38, and ERK1/2, also crosstalks with the mTOR pathway, regulating their interactions [55]. Activation of the NRF2-p62 pathway leads to increased expression of *mTOR* genes [93]. Furthermore, the activation of the P38 MAPK pathway appears to enhance *Nrf2*’s transcriptional function [94]. However, the interaction between Nrf2, mTOR, and p38 MAPK in the context of autophagy in liver cancer has not been fully elucidated.

## 4. Dual Role of Autophagy in HCC

The role of autophagy in modulating cancer progression is paradoxical and complex [6,95]. Autophagy’s role in cancer progression is complex and context dependent. It varies based on cancer type, stage, and genetics. Autophagy, as a cellular maintenance program, is cytoprotective. It removes damaged organelles, misfolded proteins, and reactive oxygen species (ROS) during stress, reducing genomic damage and the risk of mutations that could lead to cancer. 

**Figure 3:**
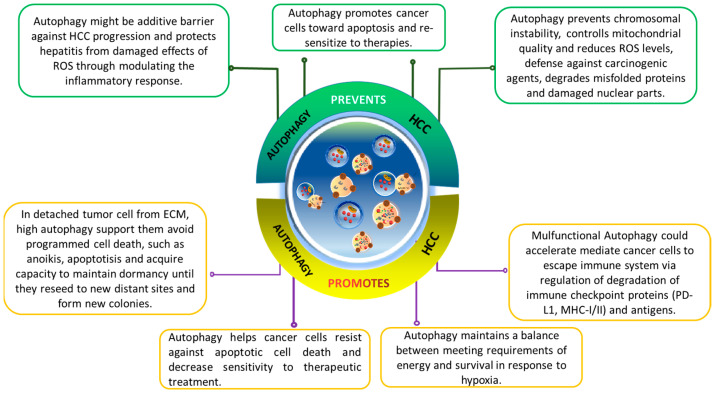
The dual-edge-sword roles of autophagy on hallmarks of cancer in hepatocellular carcinoma (HCC). Effects of autophagy on HCC are complicated and highly contextual. It ensures genomic stability to prevent mutations that promotes tumorigenesis. In tumor cells detached from ECM, autophagy helps them escape from anoikis and enables their survival. It assists the HCC cells to overcome deficient oxygen and nutrients, reprograms their metabolism, and provides resistance to cell death.

In cancer progression and metastasis, autophagy’s cytoprotective nature benefits fast-growing tumor cells, enabling them to meet increased metabolic demands for survival and rapid growth. Moreover, emerging evidence suggests autophagy plays a role in drug resistance, emphasizing its importance in cancer biology (Figure 3).

### 4.1. Autophagy Inhibits HCC Progression

In mice, when the *Beclin1* gene is partially disrupted, it reduces autophagy in vivo. This leads to an increased risk of tumorigenesis, faster cell proliferation, and the accelerated development of premalignant lesions associated with HBV. These findings indicate that Beclin1 may act as a tumor-suppressor gene and offer genetic evidence highlighting the significance of autophagy in liver cancer formation [96]. 

**Figure 4:**
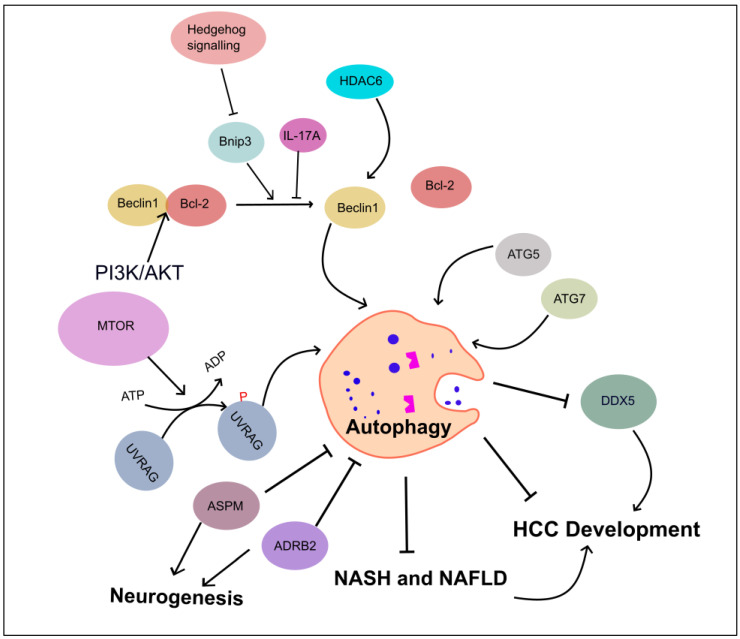
Role of autophagy-associated genes in inhibiting HCC development. This figure illustrates the regulators of autophagy in HCC development and progression. These regulatory mechanisms include Beclin1, HDAC6, autophagy-related gene proteins (ATG5 and ATG7), UVRAG, and SMURF1-mediated UVRAG ubiquitination promoting HCC. IL-17A inhibits the dissociation of Becline1/Bcl-2 complex, and PI3K/AKT enforces binding between Bcl-2 and Beclin1. ASPM and ADRB2 proteins are involved in neuronal circuit, leading to autophagy inhibition. Autophagy suppresses DDX5, leads HCC, and curtails the progression of NASH and NAFLD to HCC. Abbreviations: Adrenergic receptor beta-2 (ADRB2), Abnormal spindle-like microcephaly-associated protein (ASPM), DEAD-box helicase 5 (DDX5), and Histone deacetylase 6 (HDAC6).

Additionally, the ubiquitin-binding Histone deacetylase 6 has been shown to activate autophagic cell death in liver cancer by upregulating *Beclin1* expression through the c-Jun NH2-Terminal kinase (JNK)/Beclin1 signaling pathway [97]. Deletion of other autophagy-related genes, such as *atg5* and *atg7*, in mice models leads to the development of benign liver adenomas [98]. Moreover, liver-specific *atg7* deletion results in hepatomegaly and hepatic failure, highlighting the critical role of autophagy in maintaining liver homeostasis, the disturbance of which may contribute to the development of HCC (Figure 4).

Inactivation of the autophagy-related UV radiation resistance associated gene (*Uvrag*) enhances susceptibility to HCC development in mice [99]. Furthermore, ubiquitination of UVRAG by SMURF1 leads to the release of UVRAG from RUBCN, and phosphorylation of UVRAG by mTORC1 enforces binding of UVRAG-RUBCN to regulate its function in autophagosome maturation and HCC proliferation [100].

Increased Beclin1 dissociation from the Beclin1/Bcl-2 complex, inducing autophagy, may help trigger apoptosis and alleviate HCC. Various regulators and pathways can release Beclin1 from Bcl-2. For example, upregulating Beclin1 and disrupting the Beclin1/Bcl-2 complex by knocking down LETM1 enhanced apoptosis and autophagy while inhibiting proliferation in Huh7 and QGY-7701 cell lines [101]. However, the specific contributions of apoptosis, autophagy, or both to the inhibition of cell growth in this context remain unclear.

IL-17A, on the other hand, may prevent the dissociation of Beclin1/Bcl-2, inhibiting autophagy and promoting the viability and migration of HCC cells under starvation conditions [102]. Additionally, in HCC, the upregulation of PI3K/AKT enforces binding between Bcl-2L10 and Beclin1, accompanied by a decrease in the levels of free Beclin1, resulting in the inhibition of autophagy [103]. 

Inhibiting the Hedgehog (Hh) signaling pathway induces autophagy, not by upregulating *Atg* genes but through Beclin1 dissociation from Bcl-2 via Bnip3. This activated autophagy promotes apoptosis and hinders HCC growth. Blocking autophagy using drugs like 3-methyladenine (3-MA) or CQ or genetically through siRNA targeting Beclin1 prevents apoptosis and cytotoxicity caused by Hh downregulation [104]. 

In some cases, inhibiting autophagy can lead to the upregulation of specific proteins related to HCC progression by preventing their degradation. For instance, Heat Shock Protein 90 suppresses autophagy by downregulating the AMPK/ULK signaling pathway, which reduces the degradation of DEAD-Box Helicase 5 (DDX5). Consequently, DDX5 accumulates, promoting cell proliferation, migration, and invasion in HCC [105]. 

In addition to its effects on non-alcoholic fatty liver disease (NAFLD)-associated biological processes, autophagy may also play a role in NAFLD-associated HCC, which has been reviewed in detail previously [16]. Although direct studies examining the role of autophagy in HCC related to NAFLD using animal models are limited [15], the findings suggest that inhibition of autophagy facilitates and promotes the progression from non-alcoholic steatohepatitis (NASH) and NAFLD to HCC [106,107]. Notably, autophagy inducers, nucleolin, and Lemur tyrosine kinase 3, were upregulated after knockdown of CACHD1, a novel marker of HCC related to NASH, resulting in the significant inhibition of cell survival and proliferation [108].

In recent studies on cancer progression, the discovery of neural circuits within cancer cells has gained significant attention [109,110]. Particularly, Beta-2 adrenergic receptor (the neuron-related receptor) signaling has been found to promote HCC progression and confer resistance to sorafenib by interfering with the autophagic degradation of HIF1α, a key transcription factor involved in cancer growth and survival [111]. Abnormal spindle-like microcephaly-associated protein promotes HCC progression by activating Wnt/β-catenin signaling by antagonizing autophagy-mediated Dvl2 degradation [112].

### 4.2. Autophagy Promotes HCC Progression

Analysis of global gene expression in HCC using high-density cDNA microarrays revealed that the mRNA expression of Beclin1 was upregulated in more than 50% of liver tumor tissues and was undetectable in normal liver tissues [113,114]. Autophagy induced by Bone morphogenetic protein 4 promoted HCC cell proliferation by activating the JNK1/Bcl-2 signaling pathway and upregulating Beclin1 expression [115]. Additionally, knockdown of an oncogene, ubiquitin-conjugating enzyme E2I, downregulated the expression levels of autophagy-related proteins, including LC3A/B, Beclin1, and ATG16L1, which could potentially alleviate hepatocarcinogenesis and tumor angiogenesis [114].

Autophagy was found to be upregulated in HCC related to hepatitis virus infections, such as HBV, HCV, or HDV. In tumor tissues of HCV-HCC, compared with para-tumor tissues, the expression of LC3B, Beclin1, and ATG7 was upregulated [116]. Accumulated evidence highlights that inhibition of autophagy could enhance apoptosis signaling and consequently suppress HCC. For instance, the suppression of autophagy by knockdown of clusterin decreased proliferation and enhanced apoptosis, potentially attenuating the progression of HCC in HCV-infected individuals [116]. Around 80% of HCC patients with HBV or HCV infections exhibit increased expression of Beclin1 [117]. Moreover, increased expression of Beclin1 mRNA in HBV-related HCC could be induced by citrullinated histone H3 and may serve as an unfavorable prognostic factor for HCC patients [118]. 

In the context of HCV infection, nonstructural 5A can suppress apoptosis by inducing autophagy in a Beclin1-dependent manner, facilitating the tolerance of hepatoblastoma cells to starvation [119]. In HBV-involved HCC patients, ATG9A protein levels were increased in tumor liver tissues, but not in non-HBV HCC cases. ATG9A might be partly associated with the survival of HCC by inhibiting apoptosis [120]. As autophagy promotes the replication of HBV and HDV at different steps of their life cycle, autophagy could contribute to the progression of HCC associated with hepatitis virus infections [121]. 

Autophagy can counteract the effects of various HCC treatment approaches, including radiation and chemotherapy. In situations where radiofrequency ablation (RFA) falls short in eliminating HCC, autophagy can promote the rapid recurrence of remaining tumors via the ATP-AMPK-mTOR signaling pathway. Blocking autophagy with agents like CQ or siRNA targeting *Beclin1* or *Atg5* enhances apoptosis in HCC cells and inhibits tumor growth both in vitro and in vivo [122]. 

The monoclonal antibody CH12, targeting EGFRvII, effectively inhibits the growth of EGFRvII-positive HCC cells. However, it can also trigger additional autophagy in these cells by enhancing the binding of EGFRvII to Rubicon, leading to the release of Beclin1 from its complex. This autophagy induced by CH12 might counteract its anti-proliferative effect in certain situations. Conversely, silencing essential autophagy-related proteins promotes cell death in HCC cells [123]. 

These findings suggest that combining siRNA targeting autophagy-related genes like *Beclin1, Atg7*, or *Atg5* with autophagy inhibitors and anticancer drugs can enhance the effectiveness of HCC treatments. For example, researchers have explored the use of calcium phosphate nanoparticles to co-deliver FTY720, an immunosuppressive agent with potent anticancer properties that inhibits protective autophagy, along with Beclin1 siRNA. This approach more effectively halted HCC cell progression by suppressing protective autophagy and increasing apoptosis [124]. Combining gene therapy and anticancer drugs within nano-carriers holds promise for non-surgical tumor treatment, improving targeted delivery and treatment outcomes by modulating autophagy, a crucial factor in HCC progression and drug resistance.

In HCC treatment, targeting the deacetylation of Beclin1 could be beneficial. Acetylated Beclin1 hinders autophagy in HCC by suppressing autophagosome maturation. SIRT6, a protein involved in HCC metastasis and cell growth, promotes autophagy by deacetylating Beclin1, leading to improved autophagosome maturation and E-cadherin degradation. When autophagic gene *Atg7* is knocked down or when treated with CQ, this effect is reversed, reducing invasion and migration in HCC [125]. Additionally, stimulating sphingosine-1-phosphate (S1P) production through SPHK1 overexpression results in TRAF2 binding to Beclin1 and promoting autophagy. This leads to increased E-cadherin degradation and EMT induction in HCC cells [126]. Blocking SPHK1 activity to reduce autophagy might be a promising strategy for preventing and treating HCC.

Under hypoxic conditions, HCC cells enhance autophagy to adapt to the harsh microenvironment. YTHDF1 directly regulates the translation of ATG2A and ATG14, inducing autophagy and promoting the growth and metastasis of HCC [127]. Moreover, under oxygen/nutrient-deprived conditions, deficiency of PRMT6 (post-translational modification enzyme protein arginine N-methyltransferase 6) induces autophagy by decreasing its physical interaction with BAG5, resulting in the reduced degradation of HSC70, a well-known autophagy player. Consequently, deficient PRMT6 promotes autophagy to enhance tumorigenicity and cell proliferation in the hostile microenvironments of HCC tumors [128]. 

Inducing autophagy via the inhibition of the PI3K/AKT/mTOR pathway and the upregulation of Beclin1, or enhancing autophagy in etoposide-treated HCC cells through decreasing Beclin1/Bcl-2 interaction, is a protective and pro-survival response that suppresses apoptosis in HCC cells. Blocking the autophagy process using CQ or siRNA Beclin1 promotes apoptotic cell death [129,130]. Phosphoserine phosphatase and DEAH-box helicase 15 could induce autophagy, promote proliferation and invasion, and inhibit apoptosis in HCC cells via the AMPK/mTOR/ULK1 signaling pathway [131,132]. 

Autophagy in liver cancer stem cells (CSCs) can speed up HCC formation and progression. CD133 is a recognized liver cancer stem cell marker. Overexpressing cyclin D1 in HCC cells increases the CD133 cell proportion and inhibits p21 WAF1/CIP1 through the autophagy-miR675-PKM2 pathway. This leads to higher levels of autophagic proteins Beclin1 and LC3-II. Consequently, suppressing autophagy by silencing cyclin D1 could reduce CSC differentiation [133]. In HCC patients who had liver surgery, Beclin1 mRNA expression decreased in both HCC tumors and surrounding non-tumor tissues. Additionally, the expression of ATG8 was linked to HBV-HCC development, as low ATG8 levels in HCC tissues correlated with a poor prognosis. Furthermore, autophagy played a vital role in cancer stem cells in HCC, with the CSC marker CD90+ found to stimulate autophagy and autophagy-related genes [134].

### 4.3. Autophagy in Drug Resistance

A variety of drugs, including sorafenib, lenvatinib, regorafenib, and immune checkpoint inhibitors like pembrolizumab and nivolumab, are used in the treatment of HCC, with individualized treatment plans based on patient-specific factors and disease stage [135,136].

A significant portion of patients with advanced liver cancer fail to attain long-term benefits from drug therapy due to primary and acquired drug resistance mechanisms. This contributes to the persistently high mortality rate associated with liver cancer. Reported mechanisms of resistance to drug therapy in liver cancer include lower drug uptake, enhanced drug efflux, enhanced drug-metabolizing enzymes, alterations in drug targets, DNA repair mechanisms, the delicate balance between pro-survival and pro-apoptotic factors, adaptation to the tumor microenvironment, phenotypic transition, and more [137,138]. For more in-depth information, it would be advisable to refer to the mentioned references.

Autophagy plays a crucial role in drug resistance in HCC, as chemotherapeutic agents often induce a pro-survival form of autophagy in HCC cells [139]. Inhibiting autophagy could impair cell survival during chemotherapy treatment and enhance the growth-suppression effect of anti-cancer agents. Current evidence suggests activating Beclin1-regulated autophagy may contribute to chemo-resistance in HCC cells through different signaling pathways.

In HCC-LM3 and CSQT2 cancer cells, the protein 14-3-3ζ (gene symbol *YWHAZ*) binds and stabilizes phospho-beclin1S295, subsequently activating Beclin1-mediated autophagy to resist the cytotoxicity of chemotherapeutic drugs [140]. In Adriamycin-resistant HCC HepG2/ADM cells, the pro-survival protein Bcl-2 and the JNK2 pathway are overexpressed, and treatment with Bcl-2 inhibitors, such as ABT-737 or Apogossypolone, induces ROS and Beclin1 mediated protective autophagy by releasing Beclin1 from the cytoplasmic Beclin1/Bcl-2 complex [141]. However, the combination of ABT-737 with autophagy inhibitors, such as 3-MA, enhances cell apoptosis [142,143,144]. 

The PI3K-AKT signaling pathway is often hyper-activated in HCC and is a novel target for treatment. Development of AKT inactivation using AKT1/2 (AKTi-1/2) inhibits cell viability and proliferation and induces apoptosis. However, AKTi-1/2 induces feedback autophagy activation in HCC cells, which appears to exert pro-survival and anti-apoptotic functions. Co-treatment with autophagy inhibitors, such as 3-MA, NH4Cl, and BafA1, or siRNA of *Beclin1*, significantly potentiates HCC cell death and apoptosis induced by AKTi-1/2 [145]. Another example is the downregulation of Ankyrin-repeat-containing, SH3-domain-containing, and Proline-rich region-containing protein 2 in HCC, which promotes autophagy through interaction with Beclin1 under starvation conditions and contributes to cell survival, tumor progression, and chemoresistance in HCC [146]. 

Activating cytoprotective autophagy through the MEK/ERK pathway has been observed to counteract cell death in liver cancer cells induced by anti-liver cancer agents, such as pemetrexed, PARP inhibitors, and regorafenib [147,148,149]. Conversely, inhibiting autophagy with siRNA Beclin1 or co-treating with CQ can sensitize HCC cells to the cytotoxicity of anti-cancer drugs and promote apoptosis. Autophagy induced by downregulating the AKT/mTOR signaling pathway also contributes to drug resistance in HCC via Tumor necrosis factor-alpha-induced protein 8 (TNFAIP8) [150]. Additionally, HCC cells can resist anti-cancer drugs through the CD13/p38/heat shock protein 27/CREB axis, activating the transcription and expression of Atg7, which induces autophagy [151]. 

Knocking down Beclin1 or using 3-MA, an autophagy inhibitor, enhances the synergistic effect of vorinostat and sorafenib in HCC treatment [152]. In hypoxic conditions, where autophagy promotes cell survival and resistance to apoptosis, inhibiting autophagy can reduce chemoresistance. Beclin1-dependent autophagy activation plays a crucial role in drug resistance, and blocking autophagy could improve the effectiveness of conventional HCC therapies.

However, evidence shows that inhibition of autophagy during sorafenib treatment in HCC patients might be a culprit of the sorafenib-resistant phenomenon. Higher levels of miR-21 in sorafenib-resistant HCC cells decrease PTEN expression, sequentially activate Akt, and inhibit autophagy. In contrast, anti-miR-21 oligonucleotides can restore the sensitivity of HCC to sorafenib by promoting autophagy [153]. Another example is the combination of sorafenib and capsaicin, known to induce autophagy, which enhances the anti-tumor characteristics of sorafenib in HCC [154]. 

A genome-wide CRISPR-Cas9 screen reported that lysosomal protein transmembrane 5 (LAPTM5) is an important contributor to lenvatinib resistance in HCC. LAPTM5 promoted lenvatinib resistance by promoting autolysosome formation. The upregulation of LAPTM5, especially in HCC, was induced by DNA hypomethylation and driver mutations such as *TP53* [155]. SCAP, a cholesterol sensor, contributes to sorafenib resistance through AMPK-mediated autophagy regulation, and in particular, treatment with SCAP inhibitor lycorine can reverse acquired resistance to sorafenib [156]. 

## 5. Selective Autophagy in HCC

### 5.1. Mitophagy in HCC

Mitophagy, a specialized form of autophagy focused on removing damaged mitochondria, holds particular significance in the context of HCC for the following compelling reasons [157]: First, hepatocytes, the primary liver cells, are rich in mitochondria, vital for liver functions, emphasizing the need for their maintenance. Second, mitochondria produce ROS, which, when excessive, can lead to DNA damage and cell stress, contributing to cancer. Mitophagy selectively removes dysfunctional mitochondria, reducing this risk. Third, accumulated dysfunctional mitochondria can create a favorable environment for genetic mutations and inflammation, both implicated in cancer. Mitophagy helps remove these problematic mitochondria, reducing the likelihood of tumor formation. Fourth, HCC often develops in an inflamed liver. Dysfunctional mitochondria exacerbate inflammation by releasing ROS and mitochondrial DNA. Mitophagy moderates this response by clearing damaged mitochondria, potentially slowing cancer progression. Fifth, in advanced HCC, rapidly dividing tumor cells rely on mitophagy to maintain mitochondrial function. While disrupting this process can induce apoptosis, it can also support tumor growth by providing essential nutrients.

Emerging evidence indicates that mitophagy sustains tumor cell metabolism and provides nutrients for tumor growth and survival, thereby contributing to malignant tumor progression in HCC [158,159,160]. The PINK1-Parkin-mediated mitophagy pathway has been extensively studied and enriched in our understanding. PINK1 recruits Parkin to depolarized mitochondria to initiate mitophagy [161,162,163,164]. Additionally, accumulating evidence suggests that damaged mitochondria can be sequestered by Parkin-independent autophagy, involving autophagy mitochondrial resident receptors such as BNIP3, NIX, and FUNDC1, which directly interact with LC3 to recruit the phagophore membrane to mitochondria [165].

Mitophagy, similar to general autophagy, also plays a crucial role in the liver’s physiology and pathophysiology, including conditions like NASH-NAFLD, Alcoholic liver disease (ALD), other liver injuries, as well as HCC formation and progression. It is worth noting that mitochondria make up approximately 13–20% of the volume of the liver [166]. Selectively removing dysfunctional mitochondria through mitophagy can help reduce intracellular oxidative stress, thereby protecting against hepatic tumorigenesis. In a mouse model of HBV-HCC, PINK1/Parkin-activated mitophagy in liver cells, induced by thyroid hormone, decreased HCC incidence, and protected against the damaging effects of ROS in hepatitis [167]. Lower levels of PINK1 expression in HCC tumor tissues than in adjacent tumor parts indicate that mitophagy could be blocked in highly proliferative cells [167]. 

Moreover, mitophagy might act as an additional barrier against HCC progression by modulating the inflammatory response by clearing accumulated dysfunctional mitochondria. Recently, mitophagy regulated by FUNDC1, a mitophagy receptor protein, was shown to alleviate liver carcinogenesis by inhibiting inflammasome activation, dependent on the JAK/STAT signaling pathway [168]. 

Reports on mitophagy in drug resistance in HCC are relatively scarce. In 2016, Néstor Prieto-Domínguez and colleagues made noteworthy findings in this regard. They discovered that melatonin treatment increased the sensitivity of human HCC cells to sorafenib by inducing mitophagy and promoting the production of ROS [169]. In a subsequent study by the same research group, conducted in the same year, it was demonstrated that melatonin further enhanced the effectiveness of sorafenib in HCC cells. This enhancement was achieved by inhibiting the mTORC1/p70S6K/HIF-1α pathway and attenuating hypoxia-induced mitophagy [170]. More recently, in 2020, Wu et al. reported significant findings related to mitophagy and drug resistance in HCC. They observed that in HCC cells resistant to sorafenib due to hypoxic conditions, a hyperactivated mitophagy process was regulated by the ATAD3A-PINK1/PARKIN axis. This regulatory axis played a pivotal role in conferring resistance to sorafenib in HCC cells under hypoxic conditions [171]. These findings shed light on the complex interplay between mitophagy and drug resistance in liver cancer.

Disruption of mitophagy may lead to disrupted metabolism and increased oxidative stress, resulting in apoptosis cell death. As a result, researchers have developed natural compounds to target mitophagy in HCC treatment. Chinese herb extracts like sesamol, matrine, and alantolactone have been shown to inhibit PI3K/Beclin1 or PINK1/Parkin-mediated mitophagy, thereby promoting apoptosis in HCC cells [172,173,174]. 

Ketoconazole, a conventional antifungal drug, has also gained attention as a therapeutic approach in HCC. Ketoconazole activates mitophagy, promoting apoptosis and enhancing the anti-proliferation effects of sorafenib in HCC cells [175,176]. PINK1-dependent mitophagy can inactivate the tumor suppressor p53 located in mitochondria, thereby maintaining the survival of the hepatic CSC population, which is necessary for HCC tumorigenesis [177]. Conversely, blocking mitophagy activates p53, leading to its translocation to the nucleus, and suppressing the transcription of NANOG, a major transcription factor required for self-renewal of CSCs, thus reducing CSC survival [177]. 

Blocking cytoprotective mitophagy dependent on PINK1/Parkin during treatment with sanguinarine enhances cell death and mitochondrial apoptosis in MHCC-97H cells [178]. Inhibition of mitophagy by siRNA PINK1 in multidrug-resistant liver cancer cells promotes cell death induced by B5G1, a new betulinic acid analogue, indicating mitophagy’s impact on drug resistance [179]. The autophagy adaptor protein Optineurin is required for mitophagy activation and accelerates cell proliferation and migration of HCC [180]. Additionally, HCC development could be regulated by PH Domain Containing 1 (FGD1) via inducing autophagy and mitochondrial dysfunction [181]. Concomitant administration of sorafenib alongside glucose restriction demonstrates pronounced inhibitory effects on HCC both in vitro and in vivo, chiefly by disrupting SIAH1-mediated mitophagy pathways [182].

Overall, Mitophagy in HCC plays a dual role, depending on the tumor stage. In the early stage, it acts protectively by preventing HCC initiation, clearing damaged mitochondria, reducing DNA damage, and suppressing oxidative stress. However, as the tumor progresses, mitophagy accelerates to support high metabolic demands, promoting HCC progression.

Therefore, targeting mitophagy could be a promising approach for advanced HCC tumor cells [160]. However, enhancing mitophagy in adjacent normal cells presents a challenging future therapeutic direction. Careful consideration and further research are needed to effectively modulate mitophagy for potential therapeutic benefits in HCC treatment.

### 5.2. Other Selective Autophagy in HCC

Three specific autophagic pathways: ER-Phagy, Lipophagy, and Pexophagy emerge as crucial mechanisms with profound implications for understanding HCC development and progression. Exploring these pathways unveils their intricate contributions to liver biology and HCC, offering valuable insights into potential therapeutic strategies for combating this challenging disease. 

ER-Phagy, a process involving the selective degradation of the ER, holds significant relevance in liver function. The liver plays a pivotal role in drug metabolism, with the ER in liver cells serving as the central organelle for synthesizing crucial metabolic enzyme cytochrome P-450 (CYP). Notably, over 50% of drugs undergo oxidative metabolism by CYP within the liver [183]. To maintain cellular homeostasis, excess hepatic ER is eliminated through ER-phagy, a process initiated by the interaction between LC3 and ubiquitin-P62-decorated ER [184], This mechanism explains how ER-phagy can shield liver cells from ER stress, a vital protective function [185]. These findings suggest that ER-Phagy could serve as a regulatory component in liver drug metabolism [186], potentially contributing to drug resistance. Furthermore, it has been observed that ER stress-induced autophagy is responsible for conferring sorafenib resistance in HCC cells. Inhibition of this autophagic response could enhance HCC sensitivity to sorafenib by activating Caspase8 through cFLIP suppression [187]. Additionally, a recent discovery in 2023 sheds light on the role of ER-phagy in mediating ferroptosis in HCC cells treated with multi-targeted tyrosine kinase inhibitors [10].

Lipophagy, the selective degradation process of lipid droplets, serves a critical role in the regulation of lipid metabolism. In the liver, lipophagy plays a beneficial role in mitigating NASH by facilitating the extracellular secretion of lipids [188]. Furthermore, lipophagy acts as a protective mechanism against alcoholic fatty liver disease (AFLD) induced by short-term ethanol supplementation [189]. Moreover, lipophagy plays a multifaceted role in modulating cell death and inflammation within the context of NAFLD [190]. Notably, NAFLD and NASH represent risk factors for the development of HCC [191]. In a recent discovery, it has been observed that BNIP3, a mitochondrial cargo receptor, can reduce the growth rates of HCC by enhancing the turnover of lipid droplets at the lysosome. This process, termed “mitolipophagy,” involves the turnover of lipid droplets in association with mitochondria expressing BNIP3 [192].

Pexophagy, the selective degradation of peroxisomes, represents a crucial mechanism for eliminating damaged or excess peroxisomes. When pexophagy malfunctions, it can result in the accumulation of peroxisomes [193], which contributes to oxidative stress and inflammation [194]. Notably, oxidative stress is closely associated with the development and recurrence of HCC [195]. Remarkably, research has revealed the presence of ROS-mediated liver-specific pexophagy during extended fasting in catalase-knockout mice. This process may be implicated in hepatic cell death [196], suggesting that pexophagy could play a pivotal role in regulating cell survival in the liver. Dysregulation of this process might contribute to HCC-related cell death.

## 6. The Agents That Target Autophagy in HCC Treatment

The quest for effective HCC treatment has led researchers to explore the intricate autophagy regulation. In this field, agents are being developed to both inhibit and activate autophagy, offering a promising approach in the battle against HCC. Balancing these dual approaches is key to unlocking innovative strategies for HCC management. We will discuss both autophagy inhibitors and activators in HCC as follows (Table 1).

### 6.1. Autophagy Inhibitors

As mentioned earlier, autophagy is a complex process with multiple steps, and various components of this pathway can be potential targets for therapeutic intervention. One of the main strategies to inhibit autophagy is altering lysosome functions using inhibitors such as CQ or hydroxychloroquine (HCQ), currently the only clinically available drugs to downregulate autophagy. These drugs act as lysosomal lumen alkalizers, reducing the acidic environment within lysosomes and preventing the fusion of autophagosomes with lysosomes, thereby blocking the degradation process [197]. Notably, CQ has been shown to sensitize cancer cells to chemotherapeutic agents by blocking autophagy [198].

In the context of HCC, sorafenib and CQ combination treatment have demonstrated significant suppression of HCC growth in vitro compared to sorafenib alone [199]. Although combination therapy with CQ has been explored in various cancer types, including glioblastoma, lung cancer, breast cancer, and solid tumors, there has not been a published clinical trial investigating this combination specifically for HCC [200]. However, a phase 2 clinical trial is underway with HCC patients to evaluate the effect of this combination (NCT03037437, https://clinicaltrials.gov/, accessed on 5 September 2023), and recruitment is ongoing. This trial aims to shed light on the potential of CQ as an autophagy inhibitor in HCC treatment.

Next-generation lysosomal inhibitors, including Lys05, have shown promise in inhibiting autophagy and impairing the growth of several cancers, as demonstrated in preclinical mouse models [201]. Lys05 exhibits a higher potency as an autophagy inhibitor than HCQ, as it induces greater lysosomal deacidification [201]. In the context of HCC treatment, transarterial embolization is a common approach. However, liver cancer cells that survive after TAE can increase metastases by activating autophagy under stress conditions. In vivo results suggest that combination therapy using the autophagy inhibitor Lys05 could enhance the therapeutic effects of TAE, leading to increased tumor necrosis compared to TAE treatment alone [202].

Another class of inhibitors, such as Bafilomycin A1 (BafA1), targets V-ATPases (Vacuolar-type H (+)-ATPases) located within the lysosomal, endosomal, and other organelle membranes to decrease the acidic lysosomal environment. BafA1 has been shown to inhibit the fusion of autophagosomes with lysosomes in the rat liver cell line H-4-II-E [203]. It effectively suppresses HCC cell proliferation at nanomolar concentrations, induces caspase-independent cell death, and interferes with autophagy flux [204]. HTBPI, a phenanthroindolizidine alkaloid, has also demonstrated suppression of HCC cell growth, and in combination with BafA1, it stimulates apoptotic effects on HCC cells [178]. On the other hand, the effects of concanamycin A, another selective inhibitor of V-ATPase that increases autophagosome accumulation, have yet to be studied in HCC treatment [205].

Additionally, other potent lysosomal inhibitors, such as quinacrine, VATG-027, and VATG-032 (novel acridine and 1,2,3,4-tetrahydro acridine derivatives), have shown anti-tumor effects in vitro and preclinical mouse models [206].

In HCC, the PI3K/AKT pathway is a crucial upstream regulator of autophagy, making PI3K a potential target to interfere with autophagosome formation [207]. Among PI3K inhibitors, 3-MA, wortmannin, and LY294002 are widely used to block autophagy [208]. Furthermore, the combination of 3-methyladenine with delphinidin has shown significant inhibition of HCC by inducing necrosis [209]. Additionally, the use of LY294002 has been observed to suppress cell viability, migration, and apoptosis in HCC cells through its action on AEG-1 [210].

VPS34 is a major lipid kinase that prepares the autophagic membrane for assembly of the autophagosome [211]. SB02024, a novel highly potent selective inhibitor of VPS34, blocks autophagy in vitro and reduces the xenograft growth of two breast cancer cell lines, MDA-MB-231 and MCF-7 [212]. Treatment with an inhibitor of VPS34 affects the immune microenvironment of cancer, induces the infiltration of NK cells, CD8+ and CD4+ T effector cells into the TME, induces changes in the immune environment of melanoma and colorectal cancer, and shows a therapeutic effect [213]. In addition, in melanoma or CRC animal models, the VPS34 inhibitor SB02024 improved the therapeutic efficacy of PD-1 and PD-L1 target antibodies, which are immune checkpoint inhibitors [214]. However, treatment with a VPS34 inhibitor can cause off-target toxicity by causing loss of its role in endosome/phagocytic transport [215]; the selective targeting of autophagy in consideration of this point seems necessary for application as an anti-cancer treatment.

As such, there are many studies on the effects of VPS34 inhibitors in other cancers, but studies on the effects in liver cancer are limited. VPS34 was significantly increased in HCC tissues and liver CSCs, and the VPS34 inhibitor VPS34-IN-1 suppressed the expression of stemness genes in hepatoma cells significantly [176].

Selective inhibitors targeting only VPS34, to date, have failed in clinical treatment, and it has been mentioned that the inhibition of VPS34 alone may not exert a sufficient cancer-killing effect [216]. It seems focused, and papers on this possibility have been reported. For example, SB02024 significantly enhanced the cytotoxicity of sunitinib and erlotinib in MDA-MB-231 [212]. For details on the recent development trend of VPS34 inhibitors, it is useful to refer to the review by Liu et al. [217].

ULK1/2 inhibitors (ULKi) obstruct early autophagy and offer a promising therapeutic avenue for liver cancer. Notably, Jiang et al. conducted a virtual library screening, utilizing the X-ray crystal structure of ULK1 and its ligand binding configuration, resulting in the derivation of a ULK1 inhibitor with reported anticancer efficacy against HCC [218]. Moreover, the anticancer potential of ULK1 inhibitors extends beyond liver cancer, as evidenced by their activity in other cancer types. Specifically, ULK-100 and ULK-101 emerge as potent and selective ULKi options, impede early autophagy initiation, and trigger cell death in NSCLC [219]. Novel ULKi candidates, MRT67307 and MRT68921, have demonstrated robust in vitro performance and established tumor-suppressive activity [220]. The combination of MRT68921 and Cinobufagin effectively treat HCC [221].

Another potent and selective ULKi, DCC-3116, has shown preclinical antitumor efficacy when combined with the MAPKi trametinib [222]. A clinical phase 1/2 study is currently reported for DCC-3116, assessing safety, tolerability, clinical efficacy, pharmacokinetics, and pharmacodynamics in patients with advanced or metastatic solid tumors harboring RAS or RAF mutations (NCT04892017) [223]. Although SBI-0206965 exhibits potential as a ULKi in arresting tumor growth across diverse cancer types, including NSCLC, neuroblastoma, and renal carcinoma [224,225], its capacity to enhance the sensitivity of the anticancer drug daunorubicin in acute myeloid lymphoma [226], coupled with its inhibition of focal adhesion kinase (FAK) and AMPK, raises concerns about its specificity as a ULK inhibitor [25,224]. Recently, SBI-0206965 inhibited nutrient-starved human HCC [227]

Autophagy-related 4B (ATG4B), which regulates autophagy by promoting autophagosome formation through reversible modification of LC3, is an attractive therapeutic target because it is closely related to cancer cell growth and drug resistance [228]. Because ATG4B acts on different steps of autophagy, it is considered an alternative strategy for anti-lysosomal therapy. Therefore, drugs targeting ATG4B are more specific to the autophagy process and may play a more important role in regulating autophagy [229].

It has been shown that DC-ATG4 is an autophagy inhibitor and blocks Sorafenib-induced autophagy in HCC cells [230]. In other carcinomas, Tioconazole enhanced chemotherapeutic drug-induced cytotoxicity by inhibiting ATG4 and autophagy in colorectal cancer cell cultures and tumor xenografts [231]. Recently, as the crystal structure of ATG4B has become available, many ATG4B inhibitors have been derived through computer-aided virtual screening [229]. For example, a novel ATG4B antagonist S130 with an IC_50_ of 3.2 µM was found in a compound library through in silico screening and in vitro analysis [232]. This compound inhibited the autophagic flux and arrested the growth of the colorectal xenograft model. NSC185058, an inhibitor of ATG4B, has been reported to inhibit autophagy and inhibit the growth of Saos-2 osteosarcoma tumors. NSC185058 was also found to significantly attenuate autophagy and enhance the antitumor activity of radiotherapy in glioblastoma in a xenograft model [233].

These findings suggest that targeting autophagy-related pathways and regulators holds promise as a potential therapeutic strategy for HCC treatment.

### 6.2. Autophagy Activators

Activating autophagy through the regulation of the mTOR complex is one of the common treatment approaches for HCC, and rapamycin (sirolimus) is a well-known selective inhibitor of mTORC that induces autophagy both in vivo and in vitro [234,235]. Analogues of rapamycin, such as temsirolimus (CCI-779), everolimus (RAD001), and deforolimus (AP23573), are better therapeutic strategies against cancer due to their improved safety profile. Three ongoing randomized controlled studies are shedding light on the effectiveness of sirolimus in improving survival and preventing recurrence after liver transplantation. One of the largest trials, a multicenter phase III study involving 508 patients (Clinicaltrials.gov: NCT00355862 SiLVER), showed that using sirolimus for over 3 months post-transplant significantly reduced the risk of death compared to immunosuppression without sirolimus. Notably, patients with higher AFP levels benefited the most from additional mTOR inhibitors, experiencing improved overall survival, disease-free survival, and a reduced risk of HCC recurrence [236,237].

Furthermore, mTOR inhibitors have been investigated in combination with first-line drugs in HCC treatment to enhance efficacy. Clinical trials have shown mixed results in this regard. A phase I study with 25 incurable HCC patients in stages III-IV showed that the combination of temsirolimus and sorafenib lowered the maximum-tolerated dose (MTD) of sorafenib [238]. However, a randomized clinical trial (Clinicaltrials.gov: NCT01005199) including 106 HCC patients divided into two groups, one receiving only sorafenib and the other receiving sorafenib plus everolimus, did not show significant evidence of improved efficacy with the combination of everolimus [239]. There are several completed non-randomized phase 2 clinical trials assessing the effects and safety of combining mTOR inhibitors with sorafenib, bevacizumab, or pasireotide in advanced or metastatic HCC patients (Clinicaltrials.gov: NCT00775073, NCT01335074, NCT01488487) [240]. These trials explore the potential benefits of combination therapy and provide valuable insights for HCC treatment.

Maintaining the sensitivity of HCC cells to rapamycin has been challenging, as liver cancer cells can develop molecular mechanisms to resist rapamycin treatment. This challenge has led to the development of second-generation mTOR inhibitors, also known as rapalogs. Preclinical studies with second-generation PI3K inhibitor (BKM120) and PI3K/mTOR dual inhibitor (BEZ235) have shown that these inhibitors can better control HCC proliferation than everolimus or sirolimus. Additionally, the first results of some phase 1 clinical trials in advanced HCC with this new generation of mTOR inhibitors are now available [200]. These new inhibitors promise to improve HCC treatment efficacy and overcome resistance mechanisms observed with first-generation mTOR inhibitors.

Maintaining the sensitivity of HCC cells to rapamycin has been challenging, as liver cancer cells can develop molecular mechanisms to resist rapamycin treatment. This challenge has led to the development of second-generation mTOR inhibitors, also known as rapalogs. Preclinical studies with second-generation PI3K inhibitor (BKM120) and PI3K/mTOR dual inhibitor (BEZ235) have shown that these inhibitors can better control HCC proliferation than everolimus or sirolimus. Additionally, the first results of some phase 1 clinical trials in advanced HCC with this new generation of mTOR inhibitors are now available [240]. These new inhibitors promise to improve HCC treatment efficacy and overcome resistance mechanisms observed with first-generation mTOR inhibitors.

**Table 1:** Providing an overview of compounds and their roles as autophagy modulators HCC research. Abbreviations: chloroquine (CQ), hydroxychloroquine (HCQ).

Compounds	Autophagy Modulators	Experimental Objects	Mechanism
CQ	Inhibitor	HCC cell line: HepG2 [241]	Lysosomal Inhibitor
HCQ	Inhibitor	HCC cell lines: HepG2 and Huh7 [242]	
BafA1	Inhibitor	Rat liver cell line H-4-II-E [203]	V-ATPase Inhibitor
		HCC cell lines: (BEL7402, LO2, SMMC-7721, Huh7, and HepG2) [204]	
3-methyladenine plus delphinidin	Inhibitor	HCC cell line: SMMC7721 [209]	PI3K Inhibitors
LY294002	Inhibitor	Normal liver cell line: HL-7702 [210] HCC cell lines: SMMC-7721, BEL-7401, SK-Hep-1 and HEP-G2 [210]	
VPS34-IN-1	Inhibitor	HCC cell line: Huh7, MHCC-97H [217]	VPS34 Inhibitor
MRT68921 plus Cinobufagin	Inhibitor	HCC cell line: HepG2 and Huh7 [221]	ULK1/2 Inhibitor
SBI-0206965	Inhibitor	Normal human liver cells (L02) [227] Nutrient-starved human HCC [227]	
DC-ATG4in	Inhibitor	HCC cell lines: Huh7 and SK-HEP-1 [230]	ATG4B Inhibitor
Temsirolimus plus Sorafenib	Activator	HCC patients [238]	mTOR inhibitor
Everolimus plus Sorafenib	Activator	HCC patients [239]	

## 7. Future Perspectives

Autophagy plays a dual role in HCC, impacting its initiation, development, and progression. Its importance in managing HCC is increasingly recognized, with key pathways like PI3K/AKT/mTOR, AMPK/mTOR, MAPK, and p62-Nrf intricately involved. These autophagy-related molecules have potential as prognostic indicators and therapeutic targets in HCC. Evaluating autophagy activity in tumor tissue can provide valuable insights for patient stratification and treatment decisions.

Understanding autophagy’s role in different HCC stages, cell types, and various environmental and genetic factors is crucial for developing effective therapeutic strategies. Investigating both autophagy itself and the molecular mechanisms underlying chemotherapy and radiation resistance, especially in HCC cells, is essential. Ongoing research aims to improve overall survival rates and reduce HCC recurrence by combining autophagy modulators with anti-cancer agents, utilizing HCC cell lines, animal models, and clinical trial insights.

Efforts to discover small molecules for manipulating autophagy in HCC treatment are gaining attention. Comprehensive studies, including clinical trials, are needed to explore the feasibility of these approaches and the potential of new candidate drugs, either alone or in combination with existing medications, for combating HCC.

Similar to other cancers, targeted therapies for liver cancer must consider the timing and approach of intervention. Timing is critical due to autophagy’s role in normal tissue homeostasis and disease prevention. Strategies to control autophagy in cancer need to be developed while minimizing adverse effects, such as metastatic recurrence or neurodegeneration.

## Data Availability

Not applicable.
